# How can the sustainable development goals improve global health? A call for papers 

**DOI:** 10.2471/BLT.17.202358

**Published:** 2017-10-01

**Authors:** Christopher Dye, Shambhu Acharya

**Affiliations:** a Department of Strategy, Policy & Information, World Health Organization, World Health Organization, avenue Appia 20, 1211 Geneva 27, Switzerland.; b Department of Country Cooperation and Collaboration with the UN System, World Health Organization, Geneva, Switzerland.

Good health underpins almost everything that people want – to be free of illness, to escape poverty and hunger, to work to secure independence, to gain fulfilment through education and learning, to be treated fairly and without discrimination, and to live in a safe environment. Good health is a precondition for, an outcome and measure of, sustainable development.^1^ The United Nations *2030 agenda for sustainable development* embraces these aspirations.^2^ The sustainable development goals (SDGs)^3^ are the objectives of a programme that aims to be a comprehensive blueprint for human development.

In pursuing that aim, the 2030 agenda makes a different proposition from its predecessor, the United Nations Millennium Project (2000–15)^4^. The Millennium Project targeted major causes of illness and death in low-income countries and the millennium development goals (MDGs) focused on child and maternal mortality and major communicable diseases.^5^ In contrast, the sustainable development agenda recognizes that opportunities to improve health can be found not only in specific health interventions (principally in SDG 3), but also through social justice (SDGs 4, 5, 10, 16–17),^6^ environmental protection (SDGs 2, 6, 7, 11–15),^6^ and shared prosperity (SDGs 1, 8, 9).^6^ These three pillars of sustainable development are seen as integrated and indivisible. The 2030 agenda is not merely another proposal for mitigating causes of death; it is a vision for a better way of life. More explicitly than before, health is seen as a “state of complete physical, mental and social well-being.”^7^

The 2030 agenda recognizes that the many drivers of good health are interdependent; that they are part of a system that crosses the conceptual boundaries between professional disciplines and the administrative limits of government departments. Thus, the provision of health services and financial protection, the two essential ingredients of universal health coverage,^8^ stimulates innovation and contributes to employment and economic growth. Good health can alleviate poverty by improving people’s capacity to learn and work. By encouraging action across different segments of society the SDGs should stimulate the discovery of ways to confront today’s major challenges to health, including ageing and disabilities, non-communicable diseases, antimicrobial resistance, epidemics and health security, climate change, environmental degradation and pollution, sustainable financing, health inequities, migration, urbanization and rural poverty.

The 2030 agenda is not a finished roadmap for development; rather, it is a set of propositions that must be field-tested. Some ideas about how to improve health by modifying social, economic and environmental determinants will succeed; others will fail. In the spirit of critical evaluation, a theme issue of the *Bulletin* will examine whether and how the SDGs can serve not just as a checklist of familiar public health aims, but also as a stimulus to discover new and practical ways of accelerating gains in health.

We welcome papers on a diversity of topics related to health in sustainable development.^9^ Studies on intersectoral action by multiple stakeholders, including multidisciplinary research, Health in All Policies, One Health (at the interface between human and animal populations and the environment), integrated vector control and the role of civil society are of interest. Health systems strengthening for universal health coverage, including evaluations of people-centred^10^ health systems and ways in which major communicable disease control programmes are contributing to health systems development are within scope. Also welcomed for this issue is work on equity, ethics, fairness and human rights as core values.^11^ Linked to universal coverage, we encourage submissions on sustainable financing for health, considering new sources of funding, especially domestic finance in low- and middle-income countries, including through increased taxes on tobacco, alcohol and sugary drinks, but also co-financing of health with other sectors eg. agriculture, energy, transport, education, environment and/or industry. We would like to receive studies that evaluate the contribution made to other SDG targets by progress on four means of implementation for SDG 3 – the Framework Convention on Tobacco Control (FCTC), vaccines and immunization, access to medicines, health financing and preparedness for global health crises.

Scientific research and innovation are an integral part of sustainable development. We therefore encourage descriptions of research priorities and trends, research and development, operational and implementation research, monitoring and evaluation in all aspects of health, and new methods and technologies to manage large volumes of data. Of particular interest are disaggregated data sets that make it possible to ascertain the needs of all individuals regardless of their race, religion, ethnicity, or geographical location. 

The deadline for submissions is 1 February 2018. Manuscripts should be submitted in accordance with the *Bulletin’s Guidelines for contributors* (http://submit.bwho.org), and the cover letter should mention this call for papers.
